# Tribal Practices for Wellness in Indian Country

**DOI:** 10.5888/pcd16.180660

**Published:** 2019-07-25

**Authors:** Nancy S. Andrade, Marita Jones, Shelley M. Frazier, Chris Percy, Miguel Flores, Ursula E. Bauer

**Affiliations:** 1 National Center for Chronic Disease Prevention and Health Promotion, Centers for Disease Control and Prevention, Atlanta, Georgia; 2 Healthy Native Communities Partnership, Inc, Shiprock, New Mexico; 3 Holistic Wellness Counseling and Consultant Services, Tucson, Arizona

## Expanding Understanding of Tribal Practices that Keep People Well

For American Indian and Alaska Native tribes and communities, cultural and traditional teachings and practices are important protective factors that provide their people with strength and resilience to lead healthful lives. Tribal leaders have expressed that these practices are not widely understood by federal agencies, and often are not supported with financial and technical resources. Tribes may choose not to apply for government funding opportunities because the practices that work best for their populations are not described in the funding announcement. In February 2015, the Tribal Advisory Committee (TAC) of the Centers for Disease Control and Prevention/Agency for Toxic Substances and Disease Registry (CDC/ATSDR) recommended that CDC convene a group of knowledgeable cultural advisors to increase understanding of the role of tribal practices to support physical, emotional, and spiritual well-being. The purpose was to craft specific language to include in CDC’s funding opportunities to support implementation of these practices.

## Convening Cultural Advisors

CDC’s National Center for Chronic Disease Prevention and Health Promotion (NCCDPHP) hosted 3 convenings in 2015 and 2016, in Indian Country and at CDC headquarters. Participants were nominated by CDC/ATSDR TAC members, 1 from each of the 12 Indian Health Service regions. Healthy Native Communities Partnership, Inc (HNCP), a native nonprofit organization with extensive experience providing culturally appropriate technical assistance and consultation to tribes and Native communities, facilitated the convenings.

HNCP’s facilitation approach is uniquely designed for each group — integrating the needs and gifts of participants, the aims of the hosts, and appropriate cultural and traditional foundations for the purpose and location. TAC members, CDC staff, and HNCP clarified the rational and experiential aims for the convenings. Cultural advisors, nominated by the TAC, received personal invitations that included the need being addressed, purpose and expectations, how they were nominated, and meeting logistics.

In collaboration with CDC, the HNCP team designed a flow for each convening that built relationships and understanding among participants while integrating culture and tradition. A flexible agenda, without time constraints, showed the path the group would follow for each day. HNCP used visual and participatory processes to facilitate discussions, build trust, and create a welcoming, comfortable, and fun environment that encouraged candid discussion through an inclusive and respectful facilitation approach promoting connection to culture.

The first convening on Tribal Practices That Promote Health and Well-Being was hosted at a site operated by the Kalispel Tribe of Indians in Washington State. Cultural advisors from 10 Indian Health Service regions, 3 TAC members, 3 staff members from CDC and 1 from SAMHSA (the Substance Abuse and Mental Health Services Administration), and 3 American Indian facilitators from HNCP participated. Seated in a circle, participants were welcomed to the meeting by the host, the NCCDPHP’s director at the time. The meeting began with a prayer song to help set positive intentions. One participant, who also chaired the CDC/ATSDR TAC at the time, shared the purpose and vision of the meeting. Through a series of trust-building activities, including one in which pairs exchanged traditional gifts and introduced their partners, participants began to connect with each other on multiple levels.

The group then shared their perspectives on how culture and community contribute to health, strength, and resilience for their people. They described the many forms this takes in terms of understandings, teachings, activities, gatherings, and practices at the local level by creating a simultaneous large group drawing titled *To be strong and resilient, how and when are people connected to culture and community?* Each person explained their contributions to the drawing that served as the focus for subsequent discussions and development of understanding.

As facilitators led a conversation to organize categories of health-promoting practices, several members of the group expressed concerns about what kinds of cultural knowledge and practices were appropriate to share and which are sacred and must be protected. Trust issues with CDC as a US government agency were raised. The participant who chaired the CDC/ATSDR TAC at the time shared the background for this gathering, mentioned how it resulted from a request from the TAC, and discussed the importance to tribes of being able to include cultural and traditional approaches to wellness. He explained that they were not being asked to divulge their sacred traditional wisdom and thanked the group members for bringing up these important issues.

The first convening concluded with conversations and reflections on the day. In small groups, participants explored principles CDC should keep in mind while working together with tribes and Native communities throughout the funding process — proposal, review, implementation, and evaluation. The small groups shared their conversations, developed key themes, and identified next steps. Each participant agreed on the importance of meeting again. The group reflected on the day by expressing what went well with the convening and what could be improved for subsequent meetings, and closed with a prayer.

The second convening was held at a site operated by the Gila River Indian Community in Arizona. The convening began with a welcome, sharing of intentions, trust-building activities, establishment of group agreements, and sharing of hopes and expectations for the meeting. A visual storyboard helped to put the group’s efforts in historical perspective and to clarify the roles of tribes, CDC, the TAC, HNCP, and the cultural advisors in this work. The participants then reviewed the examples of wellness-promoting activities, practices, and teachings that had been shared during the first convening and organized these into 7 themes and strategies. The convening closed with reflections, next steps for developing Notice of Funding Opportunity (NOFO) language at another meeting, and a prayer.

The third and final convening was held at CDC headquarters in Atlanta, Georgia. The group started with a prayer and a welcome, and then honored with words of remembrance a participant who had died. After a trust-building activity, participants reviewed their work, using a Four Directions Model (Figure), focused on Listening, Dialogue, Action, and Reflection.

**Figure:**
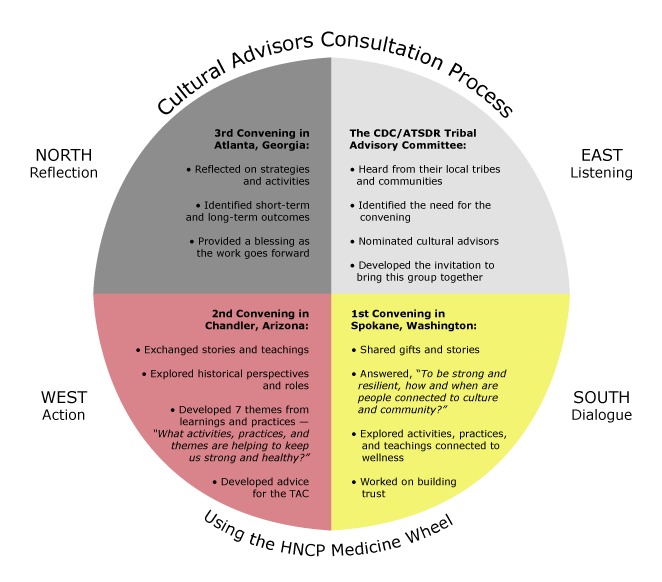
Cultural advisors consultation process. Abbreviations: CDC/ATSDR, Centers for Disease Control and Prevention/Agency for Toxic Substances and Disease Registry; HNCP, Healthy Native Communities Partnership; TAC, Tribal Advisory Committee.

Participants reviewed and revised the draft activity language under each of the 7 strategies and proposed short-term and long-term outcomes. Small work groups reviewed each of these and presented proposed changes to the full group, which came to a consensus on the revisions. After reflections on the day, the TAC chairman closed the meeting by providing a prayer for the group’s work of creating language on cultural wellness practices and blessed the documents. The Table describes the 7 strategies and corresponding activities.

**Table:** Strategies and Activities to Promote Tribal Health and Wellness

Strategies	Activities
**1. Family and community activities that connect cultural teachings to health and wellness**	Implement family-centered community activities and events working with community members and partners that teach, build upon, celebrate, and strengthen cultural and traditional practices and teachings.
	Establish or develop Native language activities for health education to promote and connect community health and Native language.
	Implement a culturally based, community-chosen activity that supports strategy 1.
**2. Seasonal cultural and traditional practices that support health and wellness**	Establish an annual community calendar of seasonal cultural and traditional events, celebrations, and activities that support and reinforce healthy practices.
	Support implementation of 1 or more seasonal and traditional cultural events, celebrations, or traditional harvest activities and engage community members and partners to make the event even healthier.
	Implement a culturally based, community-chosen activity that supports strategy 2.
**3. Social and cultural activities that promote community wellness**	Establish and/or strengthen community social and cultural activities focused on sharing cultural knowledge and practices and honoring the future through our people and youths, especially teachings of historical events, for mental and emotional well-being.
	Implement social and/or Tribal cultural activities incorporating opportunities to learn about traditional healthy food, physical activities, and lifestyle practices to enhance mental and emotional well-being.
	Implement a culturally based, community-chosen activity that supports strategy 3.
**4. Tribal, inter-tribal, governmental, and nongovernmental collaborations that strengthen well-being**	Partner with area tribes and Inter-Tribal Councils to strengthen opportunities to engage in healthy traditional, cultural, and educational activities.
	Collaborate on projects such as partnerships with community development financial institutions and other partners and sectors to increase culturally relevant economic and other opportunities.
	Implement a culturally based, community-chosen activity that supports strategy 4.
**5. Intergenerational learning opportunities that support well-being and resilience**	Establish or strengthen opportunities to encourage 2-way sharing and connect youths, adults, and elders to share knowledge about food, language, ceremonies, stories, places, technology, crafts, and play.
	Establish or strengthen opportunities for adults and elders to pass on Tribal, cultural, and other knowledge to children and young people and to other adults and elders.
	Establish and strengthen intergenerational programs that address historical trauma and that promote and enhance healing and resilience.
	Implement a culturally based, community-chosen activity that supports strategy 5.
**6. Cultural teachings and practices about traditional healthy foods to promote health, sustenance, and sustainability**	Establish or strengthen sustainable programs to gather, raise, harvest, produce, or preserve traditional healthy foods and provide those foods and beverages to individuals, families, schools, institutions, and others.
	Partner with Tribal, Inter-Tribal, governmental, and nongovernmental entities to produce and promote traditional diets, including foods and drinks to sustain health.
	Implement a culturally based, community-chosen activity that supports strategy 6.
**7. Traditional and contemporary physical activities that strengthen well-being**	Enhance, strengthen, or increase opportunities and supports for traditional and contemporary physical activity at schools, work sites, cultural and community events, and other venues.
	Enhance, strengthen, or increase traditional knowledge and history that supports traditional and contemporary physical activities at home, school, work sites, and cultural and community events.
	Build traditional or contemporary physical activity into strategies 1 through 6.
	Implement a culturally based, community-chosen activity that supports strategy 7.

The results of these convenings go beyond the specific advice and work products delivered to the TAC. At the individual level, each of the participants made significant contributions to the group and participants learned from each other, shared stories and cultural knowledge, and were heard respectfully. Participants appreciated the opportunity for honest dialogue; sensitivity toward cultural knowledge, customs, and traditions; respect and understanding for boundaries; and the ability of the group to find common ground. As the group moved through the series of convenings, participants noted that they felt positively about the teamwork, how they were able to regroup and take a different direction, the levels of intensity and passion, being reminded of what’s important, and having a vision of sustainability that reflects sovereignty. With persistence, respect, and a willingness to listen to each other, this group was able to define its own roles and complete important work together.

Results at the group and organizational level included 1) a large federal bureaucracy followed through respectfully on recommendations made by its TAC; 2) Tribal cultural advisors from different regions respectfully recognized their commonalities, differences, and boundaries; 3) the perception of federal agencies when working with Tribal cultures shifted to better understand their practices, strengths, and needs; and 4) small steps were taken toward developing trust and respect between tribes and federal agencies. Most importantly, at the level of tribes and Native communities, the work of the people who supported and participated in this important effort has the potential to revitalize cultural and traditional wellness practices leading to improvements in health, well-being, and resilience for the indigenous peoples of North America.

## Funding Tribal Practices

The CDC/ATSDR TAC concurred with the language developed during the convenings. The language was included in a NOFO that was awarded by NCCDPHP in 2018. The 3-year funding opportunity will support the tribal practices identified by the convening group and build resiliency and connections to community and culture, which over time will reduce risks for chronic disease among American Indians and Alaska Natives. The long-term goals are to reduce morbidity and mortality attributable to heart disease, stroke, cancer, and diabetes. NCCDPHP is committed to including cultural and traditional practices as fundamental elements of future programs designed to improve American Indian and Alaska Native health.

